# Blue light therapy delivered through light glasses improves sleep quality and daytime sleepiness in Parkinson's disease: A randomized trial

**DOI:** 10.1177/1877718X261468544

**Published:** 2026-08-06

**Authors:** Katarzyna Śmiłowska, Nienke M de Vries, Bastiaan R Bloem, Daniel J van Wamelen

**Affiliations:** 1 Department of Neurology, Centre of Expertise for Parkinson and Movement Disorders, Donders Institute for Brain, Cognition and Behaviour, Radboud University Medical Centre, Nijmegen, The Netherlands; 2 Department of Neurology, 5th Regional Hospital, Sosnowiec, Poland; 3 Research Center for Public Policy and Regulatory Governance, 49568 University of Silesia, Katowice, Poland; 4 Department of Human Movement Sciences, 10173 University Medical Centre Groningen, Groningen, The Netherlands; 5 Department of Neuroimaging, Institute of Psychiatry, Psychology & Neuroscience, King's College London, London, UK; 6 4616 King's College Hospital NHS Foundation Trust, London, UK

**Keywords:** Parkinson's disease, light therapy, circadian, sleep, daytime sleepiness, depression

## Abstract

**Background:**

Sleep disturbances, including excessive daytime sleepiness (EDS) and fragmented nocturnal sleep, are common in Parkinson's disease (PD) and significantly reduce quality of life. This pilot study evaluated the efficacy of a novel approach using blue light, delivered via dedicated glasses with integrated LED lights, to improve sleep and non-motor symptoms.

**Methods:**

Randomised, placebo-controlled, single-blind pilot study with a 2-week light intervention. Participants were assessed at baseline, two weeks, and five weeks. The study was designed to evaluate between- and within-group changes. Participants were randomly allocated to receive blue light therapy (n = 15) or red light placebo (n = 15), delivered via LED-integrated glasses for one hour, twice daily, given for a 2-week period. Primary outcome was improvement in sleep quality, assessed via the Pittsburgh Sleep Quality Index. Secondary outcomes included diary-based sleep outcomes, excessive daytime sleepiness, mood, anxiety, and motor symptoms.

**Results:**

There was a significant group × time effect with blue light therapy leading to better Pittsburgh Sleep Quality Index scores (*p* = 0.021). Between-group analyses showed that at two weeks a trend toward significance was observed (*p* = 0.065), while sleep quality significantly improved at five weeks compared to placebo (*p* = 0.029) with a large effect size (0.896). Excessive sleepiness improved in the blue light group (*p* < 0.001), with a reduction in clinically relevant sleepiness from 50.0% to 6.7% (*p* = 0.005).

**Conclusions:**

Blue light therapy delivered through dedicated glasses appeared to show an improvement in sleep quality and daytime sleepiness in individuals with PD. Blue light therapy offers a promising alternative to traditional light therapy utilising lower light intensities and eliminating the need for light boxes.

## Plain language summary

Blue light glasses may help people with Parkinson's sleep better and feel less sleepy during the day: results from a randomized study.

Sleep problems are very common in people with Parkinson's disease. Many experience difficulty staying asleep at night and feel very sleepy during the day. These problems can greatly affect daily functioning, mood, and overall quality of life. Light therapy is sometimes used to help regulate sleep patterns, but traditional devices can be inconvenient because they require sitting in front of a bright light box.

This study tested a new approach: using special glasses that shine blue light from small LED lights built into the frame. The aim was to see whether wearing these glasses for one hour, twice a day, could improve sleep and daytime sleepiness in people with Parkinson's disease. The study compared blue light glasses with red light glasses, which served as a placebo. Participants used the glasses for two weeks and were assessed at the start of the study, after two weeks, and again after five weeks.

The results showed that people who used the blue light glasses slept better than those using the red light glasses. Sleep quality continued to improve even after the light sessions had ended. Daytime sleepiness also improved significantly: the proportion of participants with clinically significant sleepiness fell from 50% to just 7% after using blue light.

These findings suggest that blue light therapy delivered through wearable glasses may offer a practical, low-intensity alternative to standard light therapy. Although this was a small pilot study, the results are promising and support further research to better understand who might benefit most and how blue light therapy can be used in everyday life to support people with Parkinson's disease.

## Introduction

Parkinson's disease (PD) is a neurodegenerative disorder characterised by the coexistence of motor and non-motor symptoms.^1,2^ Among the non-motor manifestations, sleep disturbances are highly prevalent, affecting up to 90% of individuals with PD, and significantly reducing quality of life and daytime functioning.^3–5^ Sleep impairment may refer to diurnal or nocturnal symptoms including sleep fragmentation, difficulties with sleep initiation, but also excessive daytime sleepiness (EDS). The underlying biology of sleep disorders in PD is heterogenous. Despite this heterogeneity, it is likely that neurodegenerative changes within central sleep regulatory areas, particularly the hypothalamus, play a crucial role in the pathophysiology of sleep and circadian rhythm disruptions observed in PD.^6^

The suprachiasmatic nuclei (SCN), located in the anterior hypothalamus, serve as the central circadian pacemaker, regulating endogenous physiological cycles with an approximate 24-h period. These are likely perturbed in PD.^6^ The circadian cycles are synchronised to the environmental light or dark and to social activity cycles by zeitgebers (cues indicating environmental time).^7^ Light represents the most effective zeitgeber of the circadian system. Supplementary exposure to light has been shown to have beneficial effects on sleep quality and daytime vigilance in healthy older people and patients with dementia.^8,9^ Stimulated by these findings, bright light therapy (BLT), utilising the whole spectrum of visible (white) light, was introduced as a measure to improve circadian rhythmicity, mood and sleep disorders in PD.^10^ BLT is considered a safe non-pharmacological treatment option, with only minor side effects. The therapeutic effects are attributed to its role as a zeitgeber, helping to reset circadian rhythms. This resetting is reflected by shifts in serum melatonin concentrations, which play a crucial role in regulating sleep-wake cycles.

BLT has been investigated as a non-pharmacological intervention for managing non-motor symptoms in PD. Exposure to light intensities ranging from 1000–6000 Lux for durations of 30 to 90 min, administered either in the morning or evening, has been shown in some studies to improve mood, sleep quality and motor function in individuals with PD.^11–15^ Nonetheless, the efficacy of BLT as treatment in PD has yielded conflicting results.^13,16,17^ Traditional methods of delivering this therapy, i.e., light boxes, can be impractical for some patients, potentially affecting adherence and overall effectiveness.^18^ Moreover, as already alluded to, BLT encompasses the whole spectrum of visible light and, as such, often requires higher light intensities to achieve therapeutic effect. Emerging evidence suggests that targeting specific wavelengths, particularly in the blue spectrum (approximately 460–480 nm) may offer a more efficient approach in entraining the SCN compared to monochromatic light with a wavelength around 555 nm.^18–20^ Moreover, blue light around 460 nm wavelength was more effective in phase-shifting circadian output and necessitated a shorter duration and lower irradiance to achieve this effect compared to exposure to white light.^19^

In this pilot study we propose a novel approach to light therapy using not only blue light as opposed to white light but also introducing a novel method of delivery. Specifically, we have tested dedicated light therapy glasses with integrated LED lights that emit blue light with a 468 nm wavelength that more specifically targets retinal melanopsin and the retinohypothalamic tract; this is more likely to effectively reset the SCN in individuals with PD compared to traditional BLT.^21,22^ In line with our previously published data,^18,20^ we expected that replacement of light boxes with the more feasible glasses would positively influence compliance.^18^ Finally, the results might give further insights into the mechanisms underlying circadian disruption in PD. The primary aim of the study was to show improvement of sleep quality, measured through the Pittsburgh Sleep Quality Index (PSQI), in individuals with PD receiving blue light therapy compared to placebo. Secondary aims included improvements in diary-based sleep quality measures, excessive daytime sleepiness, anxiety and depressive symptoms, and motor scores in those treated with blue light therapy compared to placebo.

## Methods

This was a placebo controlled single-blind multi-center study performed in the Netherlands and in Poland. It was registered on the Dutch Central Committee on Research involving Human Subjects trial register on October 19, 2017 (NL-OMON44444) and received ethical approval from METC Oost-Nederland (reference number: 2017-3622) for participants recruited in the Netherlands and the Silesian Medical Chamber (reference number: 17/2019) for participants recruited in Poland. Enrolment of participants began on January 10, 2018. All participants provided written informed consent, and all study procedures were performed in accordance with the declaration of Helsinki.

Participants were recruited from the neurology outpatient clinic at the Radboudumc (Nijmegen, the Netherlands) or the neurology outpatient clinic at the Silesian Center of Neurology in Katowice (Poland) between January 2018 and January 2023, with an interruption between March 2020 and January 2022 due to the COVID-19 pandemic. Potentially eligible participants visiting these outpatient clinics were asked by their treating physician to participate.

Inclusion criteria included: 1) diagnosis of PD according to UK Parkinson's Disease Society Brain Bank Clinical Diagnostic Criteria^23^; 2) Hoehn & Yahr stage 2 to 4^24^; 3) ability to obtain written informed consent for participation; 4) age ≥30 years; 5) score of ≥5 on the Pittsburgh Sleep Quality Index score (PSQI)^25^ or EDS defined by an Epworth Sleepiness Scale (ESS) score of ≥7^26^; and 6) stable PD treatment regime for at least 30 days prior to screening. We acknowledge that the PSQI threshold of ≥5, rather than the more commonly used cutoff of >5 (i.e., ≥6), was applied for inclusion. This decision was pragmatic, reflecting the pilot nature of the study and the need to maintain adequate recruitment in a population with multiple comorbidities. Participants scoring exactly 5 on the PSQI were considered to have borderline sleep impairment and were deemed appropriate for inclusion given the additional requirement for daytime sleepiness or subjective sleep complaints. Exclusion criteria were: 1) significant cognitive impairment as defined by a Montreal Cognitive Assessment (MoCA) score <24; 2) clinically significant psychiatric illness, including psychosis, major depressive disorder, or a lifetime history of suicidal attempt (active attempt, interrupted attempt or aborted attempt); 3) antidepressant treatment for less than three months prior to screening; 4) participation in other, interventional, research studies; 5) known conditions associated with sleep disorders other than PD, such as a known diagnosis of obstructive sleep apnoea syndrome and eye-related diseases (e.g., cataract, glaucoma, blindness); 6) any other condition that in the opinion of the investigator might have interfered with interpretation of the study results or constituted a health risk for the participant; 7) history of malignancy or migraine ≤5 years before screening; and 8) current use or use within three months prior to the screening of hypnosedative or stimulant drugs, e.g., benzodiazepines, dexamphetamine, methylphenidate, or melatonin.

After successfully completing screening, participants were allocated randomly to receive either blue light therapy (intervention) or spectacles with red lenses but without light emission (placebo). Randomisation was performed by an independent researcher with allocation to either group performed in a 1:1 manner using a computerised sequence (block randomization with varying block size) constructed by an independent statistician. Participants were blinded to the allocation and neither prior nor during their participation, were participants informed about the meaning of the colour of glasses concerning the intervention and placebo.

Dedicated glasses (Propeaq^TM^) were used for both the intervention and placebo group, consisting of a pair of light weight glasses with interchangeable lenses, which were either of red or blue colour.^18^ The intervention group received the glasses with blue lenses, and the placebo group identical glasses with red lenses. Each pair of glasses had LED light sources integrated into the frame, emitting blue light (λ = 461 ± 10 nm, spectral irradiance of 22.34 µW/cm2/melanopic EDI of 114.38 lx at the cornea of the eyes),^27^ which was only activated for the intervention group. For the placebo group the LED function was not used and could not be turned on by participants.

As this was a single-blind study, blinding success was not formally assessed through a post-study questionnaire. We acknowledge this as a limitation. The perceptible difference in lens colour between the blue (active) and red (placebo) glasses may have introduced expectancy effects, and we cannot exclude the possibility that participants inferred their group allocation. However, participants were explicitly not informed in any way about the significance of the type of colour in relation to the intervention or placebo, either at enrolment or during the study period, in an effort to minimise such bias.

Participants had to wear dedicated light therapy glasses for one hour twice daily (total two hours each day). During the first daily session participants wore the glasses for one hour after they woke up in the morning and during the second session, they wore the glasses for one hour starting two hours before preferred bedtime. Placebo consisted of red light therapy using the same glasses as the blue therapy but with red coloured glass/lenses instead of blue coloured glass/lenses. Duration and timing of placebo treatment was identical to the blue light protocol, requiring participants to wear the glasses twice daily for one hour (total two hours each day). The twice daily regime was based on previous study designs using BLT.^11–15^ Each participant used either the active or placebo treatment for a period of two weeks. All participants were invited for a study visit to perform study related assessments at baseline, two weeks (coinciding with the end of treatment) and at five weeks. All assessments, including the provision of the light therapy glasses, were performed by the same movement disorders neurologist (KS).

At baseline, week 2, and week 5 visits participants underwent the following assessments related to the study outcomes. 1) Visit 1 (baseline): collection of demographic and co-morbidity data, collection of PD related demographic data. Physician-completed study assessments included the Unified Parkinson's Disease Rating Scale (UPDRS), Montreal Cognitive Assessment (MoCA), and Clinical Global Impression of Change scale (CGI-C). Participant completed outcomes included the PSQI, Epworth Sleepiness Scale (ESS), and Hospital Anxiety and Depression Scale (HADS). 2) Visit 2 (at two weeks): completion of the UPDRS, PSQI, ESS, and HADS. 3) Visit 3 (at five weeks): completion of the UPDRS, CGI-C, PSQI, ESS, and HADS. During the 2-week treatment period, participants in each group additionally kept a sleep diary for each day. Sleep diaries recorded subjective sleep duration, sleep latency, number of awakenings at night, time spent awake at night, sleep quality, and feeling refreshed after waking up in the morning.

The primary outcome was improvement of sleep quality, as measured using the PSQI, at five weeks. Secondary outcomes included diary-based sleep quality measures, and a range of other non-motor symptoms (excessive daytime sleepiness, mood, and anxiety) and on motor symptoms.

As we envisaged this study as a pilot to be used for sample sizes calculations for future larger studies, we did not perform formal sample size calculations and included 15 participants with PD per group as a pragmatic approach, assuming a large effect size for the primary outcome.^18^ For the primary outcome (i.e., changes in PSQI scores after 5 weeks), for between-group changes over time, a linear mixed-effects model was used, and for within-group changes, given the non-normal data distribution (tested through the Kolmogorov-Smirnov test), Friedman's test was used. In addition, we performed post-hoc analyses for each time point (baseline, week 2, and week 5) to determine differences between groups. For all secondary outcomes (changes in diary outcomes, UPDRS, ESS, HADS, and CGI scores) the same procedure as for the primary outcome was followed. For baseline demographics we used the Mann-Whitney U test to determine intergroup differences; proportional differences were assessed using the Chi-Square test. Finally, we determined effect sizes for each outcome that showed statistically significant between-group differences. A correction for multiple testing using formal Bonferroni correction for each family of comparisons was used, dividing the limit of significance (set at 0.05) by the number of comparisons. All analyses were performed using SPSS version 29.

Anonymized data not published within this article will be made available by request from any qualified investigator. The study protocol and statistical analysis plan are available in eSAP 1.

## Results

**Figure 1.**
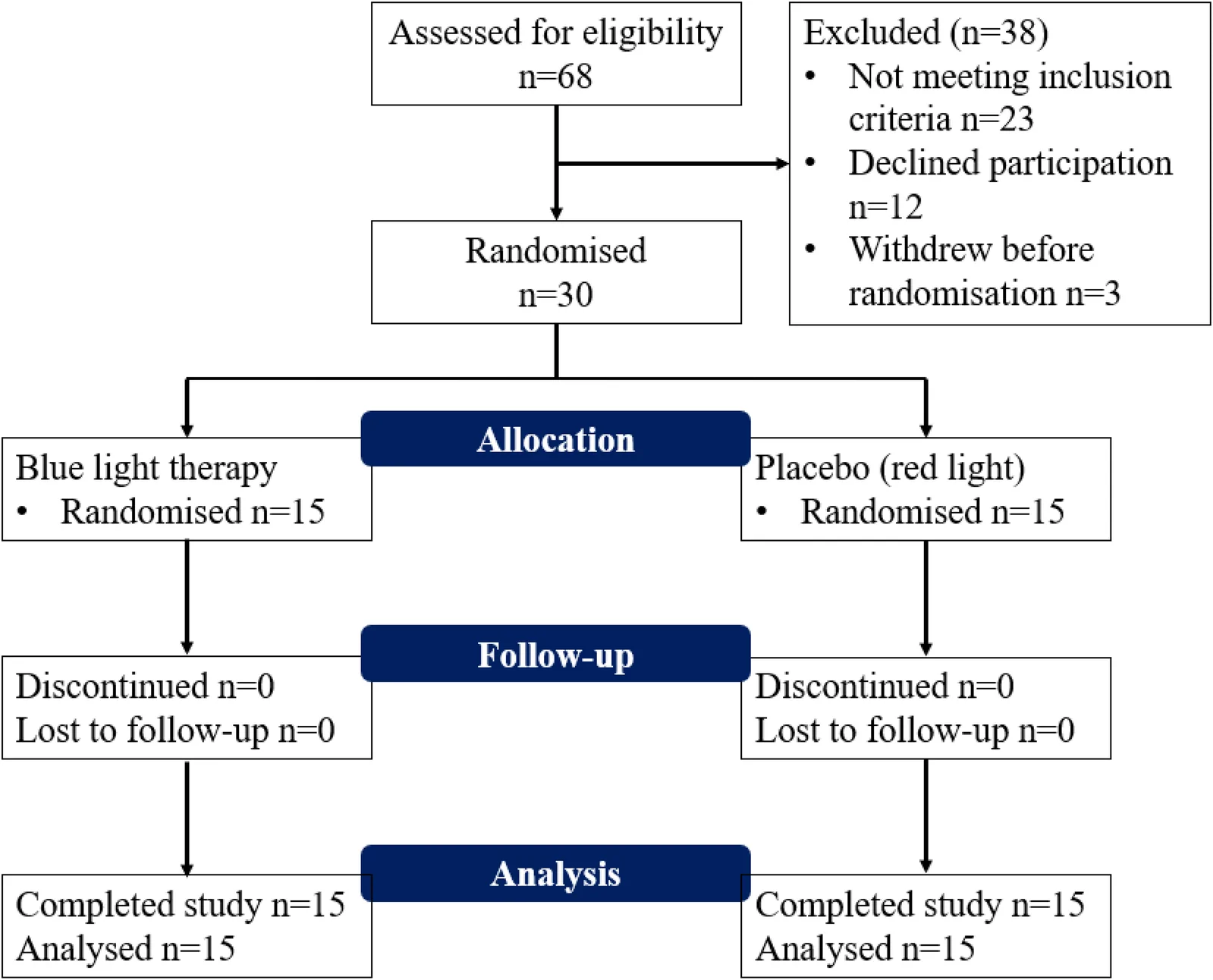
Allocation of participants.

An overview of treatment allocation is provided in Figure 1. We did not observe demographic differences between participants allocated to receive blue light therapy (active intervention) or placebo (*p* ≥ 0.056; Table 1).

**Table 1.** Baseline demographics.

	Blue light therapy (n = 15)	Placebo (n = 15)	p*
Sex (M/F)	8/7	7/8	0.715^a^
Age (years)	71.73 ± 4.06	70.80 ± 6.13	0.838^b^
Disease duration (years)	7.87 ± 2.88	7.93 ± 1.79	0.595^b^
LEDD (milligram)	1288.67 ± 341.82	1216.67 ± 468.64	0.870^b^
UPDRS Part I	9.93 ± 2.31	9.13 ± 2.95	0.412^b^
UPDRS Part II	7.07 ± 2.25	9.27 ± 3.45	0.056^b^
UPDRS Part III	55.33 ± 12.51	56.80 ± 16.49	0.902^b^
UPDRS Part IV	4.53 ± 1.19	4.53 ± 1.25	1.000^b^
HY stage	3.0 (2.0–3.0)	2.0 (2.0–3.0)	0.464^a^
PSQI	12.27 ± 2.87	11.00 ± 2.93	0.217^b^
ESS	10.60 ± 2.17	11.33 ± 2.47	0.436^b^
HADS depression	8.73 ± 3.11	9.07 ± 3.15	0.775^b^
HADS anxiety	3.60 ± 2.35	4.13 ± 2.17	0.486^b^
MoCA	25.60 ± 1.92	25.13 ± 2.23	0.567^b^

Data presented as mean ± standard deviation, absolute number, or median (25^th^-75^th^ percentile).

M: male; F: female; LEDD: Levodopa Daily Equivalent Dose; UPDRS: Unified Parkinson's Disease Rating Scale; HY: Hoehn and Yahr; PSQI: Pittsburgh Sleep Quality Index; MoCA: Montreal Cognitive Assessment; ESS: Epworth Sleepiness Scale; HADS: Hospital Anxiety and Depression Scale

* corrected for multiple testing using Bonferroni Holmes correction (level of significance set at 0.05/14 = 0.0036)

a Chi-Square test

b Mann-Whitney U test.

**Figure 2.**
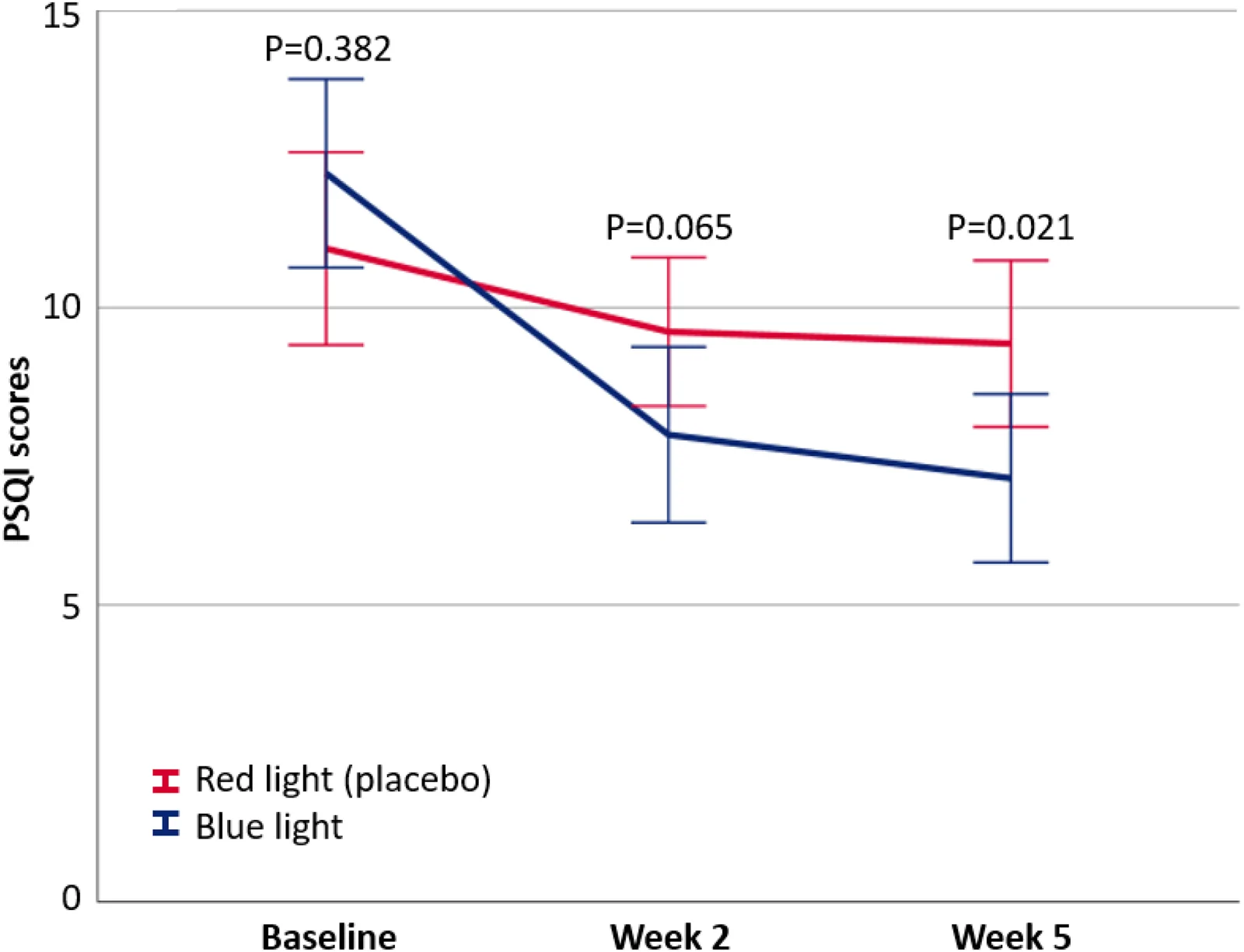
Changes in Pittsburgh Sleep Quality Index (PSQI) scores in people with Parkinson's disease following two-week treatment with blue light therapy delivered through LED light spectacles or placebo. Bold lines represent means and error bars 95% confidence interval. Group × time effect was significant (*p* < 0.001; mixed effects model).

The linear mixed-effects model showed that a significant main effect of time was present (*p* < 0.001), showing improvement of sleep quality, as well as a significant group × time interaction (*p* < 0.001), reflecting differential changes between groups and showing that intervention with blue light led to a significant improvement in sleep quality over time relative to placebo (red light) (Table 2; Figure 2). The effect size of the two-week treatment with blue light therapy was 0.896 (large effect) at the 5-week timepoint, whereas for placebo this was 0.450 (small effect). Sleep diaries, used as a secondary outcome, also showed a significant improvement in nearly all domains in the active treatment group (Supplementary Table 1). Notably, greater improvements of total sleep time, sleep latency, and sleep quality were present for the intervention group compared to the placebo group.

**Table 2.** Changes in Pittsburgh Sleep Quality Index scores in people with Parkinson's disease following two-week treatment with blue light therapy and placebo.

	Blue light (n = 15)	Placebo (n = 15)	Group differences (p)
Baseline	12.27 ± 2.87	11.00 ± 2.93	0.382
Week 2	7.87 ± 2.67	9.60 ± 2.26	0.065
Week 5	7.13 ± 2.56	9.40 ± 2.53	0.029
Change over time (p)	*p* < 0.001	*p* < 0.001	Group × time effect *p* < 0.001

Values represent mean ± standard deviation. Abbreviations: a: Wilcoxon signed rank test; b: Friedman's test post hoc.

More participants receiving blue light therapy compared to placebo improved, as judged by the CGI-C scores. Specifically, after two weeks, 40.0% of participants in the active intervention group were judged as much improved (33.3% in the placebo group), while 46.7% had improved very much (26.7% in the placebo group). This difference was not statistically significant (*p* = 0.363). However, after five weeks, this difference was statistically significant, with 53.3% of participants in the active intervention group being judged as much improved (20% in the placebo group), and 40.0% as very much improved (0% in the placebo group) (*p* < 0.001; Supplementary Table 2).

We also observed improvements in several secondary outcomes. Notably, there was an improvement in ESS scores with statistically significant differences between groups at week 2 (*p* < 0.001) and week 5 (*p* = 0.001) (Table 3). Overall, group × time showed a significant effect (*p* < 0.001). Also within groups improvements over time were observed, in both the placebo group (*p* = 0.018) and blue light therapy group after two (*p* < 0.001) and five weeks (*p* = 0.001) (Table 3). Effect sizes of improvement were 0.918 (large effect) for the blue light intervention and 0.268 (small effect) for placebo. In addition, the proportion of participants with clinically relevant excessive daytime sleepiness (ESS scores >10)^26^ decreased from 50.0% to 6.7% after five weeks in the active intervention group, compared to no changes in the placebo group (62.5% at baseline and 53.3% after five weeks) (*p* = 0.005).

**Table 3.** Changes in secondary outcome measures in people with Parkinson's disease following two-week treatment with blue light therapy and placebo.

	Placebo (n = 15)	Blue light (n = 15)	Group differences (p)
**ESS**			
*Baseline*	11.33 ± 2.47	10.60 ± 2.17	0.468
*Week 2*	10.13 ± 2.62	6.67 ± 1.40	<0.001
*Week 5*	10.93 ± 2.28	8.33 ± 1.72	0.001
**Change over time (p)**	0.018	<0.001	Group × time *p *< 0.001
**HADS depression**			
*Baseline*	9.07 ± 3.15	8.73 ± 3.11	0.958
*Week 2*	8.80 ± 3.26	6.33 ± 1.76	0.015
*Week 5*	8.60 ± 3.04	6.93 ± 2.22	0.098
**Change over time (p)**	0.266	<0.001	Group × time *p* = 0.002
**HADS anxiety**			
*Baseline*	4.13 ± 2.17	3.60 ± 2.35	0.468
*Week 2*	4.20 ± 2.01	3.00 ± 1.60	0.116
*Week 5*	3.60 ± 1.99	3.13 ± 1.81	0.624
**Change over time (p)**	0.027	0.055	Group × time *p* = 0.709
**UPDRS part I**			
*Baseline*	9.13 ± 2.95	9.93 ± 2.31	0.412
*Week 2*	7.60 ± 2.95	5.00 ± 1.31	0.005
*Week 5*	8.87 ± 2.59	8.33 ± 1.92	0.653
**Change over time (p)**	0.040	0.15	Group × time *p* = 0.625
**UPDRS part II**			
*Baseline*	9.27 ± 3.45	7.07 ± 2.25	0.056
*Week 2*	8.80 ± 3.43	6.27 ± 1.67	0.021
*Week 5*	9.13 ± 2.92	6.60 ± 2.10	0.019
**Change over time (p)**	0.030	0.80	Group × time *p* = 0.158
**UPDRS part III**			
*Baseline*	56.80 ± 16.49	55.33 ± 12.51	0.929
*Week 2*	55.40 ± 15.66	52.00 ± 10.19	0.631
*Week 5*	54.87 ± 15.22	50.93 ± 9.86	0.486
**Change over time (p)**	<0.001	<0.001	Group × time *p* = 0.111
**UPDRS part IV**			
*Baseline*	4.53 ± 1.25	4.53 ± 1.19	0.958
*Week 2*	4.33 ± 1.23	4.13 ± 1.06	0.683
*Week 5*	4.20 ± 1.27	3.73 ± 0.96	0.363
**Change over time (p)**	0.022	<0.001	Group × time *p *< 0.001

Values represent mean ± standard deviation.

Other improvements included lower HADS depression scores in participants receiving blue light therapy (group × time *p* = 0.002) with a moderate effect size (0.557), which was seen at week 2 (*p* = 0.015), but not at week 5 (*p* = 0.098) (Table 3). In both groups, we observed a small but statistically significant improvement in UPDRS part IV scores (Table 3; *p* < 0.001), but not for UPDRS parts I to III, nor for HADS anxiety scores (Table 3).

Four adverse events (AEs) occurred during the study. In the active intervention group, one participant experienced a headache. In the placebo group, one participant reported a headache, another experienced dizziness, and the third participant had itchy eyes. All AEs were mild and possibly related to the intervention. Each AE resolved spontaneously within a maximum of one day without further complications. There were no significant between-group differences in the occurrence of AEs (*p* = 0.607). No serious AEs occurred.

## Discussion

In this randomised, single-blind controlled pilot study, we assessed the efficacy of blue light therapy, delivered through dedicated glasses with integrated LED lights, on sleep quality and secondary non-motor outcomes in individuals with PD. Participants receiving the active blue light intervention demonstrated clinically meaningful improvements in sleep quality compared to placebo, as well as clinically meaningful improvements in daytime sleepiness and smaller effects on depressive symptoms. The blue light therapy was well-tolerated, with only minimal adverse events, indicating its feasibility as a treatment option for individuals with PD. This novel treatment delivery method offers a practical alternative to traditional light therapy approaches, eliminating the need for light boxes and allowing for effective treatment at lower light intensities.

The biological plausibility of blue light therapy in PD rests in part on the well-established pathological involvement of the hypothalamic suprachiasmatic nucleus (SCN) in this condition. The SCN, located in the anterior hypothalamus, functions as the master circadian pacemaker, coordinating endogenous 24-h physiological rhythms through its direct retinal input via the retinohypothalamic tract. Histopathological studies have demonstrated that the SCN is affected by neurodegenerative changes in PD, including α-synuclein pathology and neuronal loss, with post-mortem studies showing significantly reduced neuronal density and neuropeptide expression, particularly vasoactive intestinal polypeptide (VIP) and vasopressin, within the SCN of individuals with PD compared to controls.^6^ These changes are thought to directly impair the amplitude and timing of circadian output, contributing to the fragmented sleep-wake cycles and excessive daytime sleepiness so frequently observed in PD. Excessive daytime sleepiness in PD is multifactorial, reflecting not only nocturnal sleep fragmentation but also intrinsic dysregulation of wake-promoting circuitry, including orexinergic (hypocretin) neurons of the lateral hypothalamus, which are also depleted in PD. Against this background, the rationale for targeted photic stimulation via blue light, which acts specifically through melanopsin-containing intrinsically photosensitive retinal ganglion cells (ipRGCs) projecting directly to the SCN via the retinohypothalamic tract, is compelling. Blue light at approximately 460–480 nm is uniquely potent in activating ipRGCs and thereby stimulating SCN entrainment, potentially compensating for the attenuated endogenous circadian drive in PD. The substantial improvements in both sleep quality and excessive daytime sleepiness observed in our active treatment group may therefore reflect, at least in part, a partial restoration of circadian amplitude through enhanced photic zeitgeber input to a structurally compromised but functionally plastic SCN.

Both the active blue light therapy and placebo treatment exhibited significant reductions in PSQI scores, indicating improvements in sleep quality. However, the active intervention group demonstrated a more substantial improvement, accompanied by a large effect size, suggesting a clinically meaningful enhancement in sleep quality. These findings extend existing research indicating not only that light therapy can positively influence circadian rhythm regulation and sleep quality in individuals with PD,^10–15,19,28^ but that there seems to be an additional effect of blue light. Moreover, our findings expand those previously made with the same device in an open label study in PD, and which also suggested the feasibility of blue light therapy delivered via dedicated glasses.^18^

In addition to improvements in the primary outcome, we also observed improvements in several secondary outcomes. Specifically, participants receiving blue light therapy exhibited substantial reductions in excessive daytime sleepiness, as measured by the ESS, at both the two-week and five-week assessments. Moreover, we observed a reduction in the proportion of participants with clinically relevant excessive daytime sleepiness in the active treatment group, reducing this from 50% of participants to almost none over five weeks. This contrasts with the findings of a recent meta-analysis, showing that BLT did not improve sleepiness scores, measured through ESS, across five studies with 92 participants, although there was a minor improvement in post-BLT sleepiness compared to pre-BLT sleepiness levels.^29^ However, in several of these studies the study population may have included mostly participants with normal levels of daytime sleepiness, leaving little room for further improvement (a potential floor effect), as pre-intervention ESS scores were often within the normal range.^10^

It is important to acknowledge the limitations of our study. First, we studied a relatively small group of individuals with PD and the duration of the study was short. However, this was partially offset by the enrichment of participants, who were selected for having a reduced sleep quality and excessive daytime sleepiness, based on previously determined cut-off scores. As a result, we observed large effect sizes for the improvement in the primary and some secondary outcomes after blue light therapy. On the other hand, we observed no convincing changes in depressive symptoms or motor symptoms, as opposed to several previous studies employing BLT, perhaps because of the shorter treatment duration in our study (two weeks). Other limitations include the single-blind nature of the study, where participants but not investigators were blinded to the intervention type, which may have introduced some degree of bias in the assessment of outcomes. Nonetheless, blinding in non-pharmacological studies remains challenging.^30^ It is important to acknowledge that appropriate control conditions are crucial in any trial to accurately determine efficacy and avoid inflated effects due to placebo responses or expectancy biases. There is quite consistent evidence that placebo effects in individuals with PD are pronounced and linked to measurable neurobiological changes, including dopamine release in the dorsal striatum, associated with motor improvement, and the ventral striatum, linked to expectancy of reward.^31,32^ However, these mechanisms have only been explored in pharmacological contexts and in relation to motor symptoms. It remains unclear whether similar neurobiological placebo pathways are activated in non-pharmacological interventions, e.g., light therapy and exercise, or have an effect on non-motor outcomes. Further research should therefore explore the effects following more prolonged exposure to blue light therapy, as well as optimising control group designs. This should also include the long-term feasibility of the protocol described here with two sessions of light therapy per day. Future studies should additionally incorporate circadian rhythm measures—such as core body temperature timing and amplitude, as well as melatonin rhythm timing and amplitude—to better understand the physiological basis of the improvements in sleep and other outcomes observed following blue light therapy. A limitation of the current study is the exclusive reliance on subjective sleep measures. The absence of objective sleep assessments, such as polysomnography or actigraphy, may limit the conclusions that can be drawn regarding the physiological effects of blue light therapy on sleep architecture and circadian parameters. Furthermore, objective circadian biomarkers, such as dim-light melatonin onset, core body temperature rhythm, and 24-h actigraphy-derived rest-activity profiles, would have provided mechanistic insight into the circadian effects of blue light. Future studies should incorporate these measures as primary or key secondary outcomes to substantiate the subjective improvements reported here. An additional limitation concerns the choice of sleep outcome measure. The PSQI, while widely used and validated in the general population, has limited specificity for PD-related sleep disturbances. Furthermore, the PSQI instructs respondents to reflect on the preceding month, making it suboptimal for detecting changes over periods as short as two to three weeks. Nonetheless, the PSQI has clear, widely used cut-offs for identifying poor sleep and captures broader, non-motor contributors such as mood and general sleep disturbances. It might also be more sensitive in early or mild PD, where disease-specific nocturnal symptoms may be limited. Future trials may consider employing the PDSS-2 as a primary outcome, supplemented by actigraphy and sleep diaries.

The perceptible difference between blue and red lenses may have allowed some participants to infer their group allocation, potentially inflating treatment effects through expectancy. Formal blinding assessment was not performed, and we acknowledge this as a methodological limitation. Notably, both groups showed improvement, with a substantially greater and clinically meaningful improvement in the active group, and effect sizes were large, suggesting that the observed improvements are unlikely to be attributable to a placebo response alone. Nonetheless, future studies should incorporate validated blinding assessment tools and consider additional active control conditions to further disentangle specific from non-specific effects.

In conclusion, our findings suggest that blue light therapy may be a safe and effective intervention for enhancing sleep quality and daytime sleepiness in individuals with PD. Further research with extended follow-up is warranted to confirm these findings and to explore the long-term compliance as well as effects of blue light therapy on other non-sleep-related symptoms of PD.

## Supplemental Material

sj-docx-1-pkn-10.1177_1877718X261468544 - Supplemental material for Blue light therapy delivered through light glasses improves sleep quality and daytime sleepiness in Parkinson's disease: A randomized trial Supplemental material, sj-docx-1-pkn-10.1177_1877718X261468544 for Blue light therapy delivered through light glasses improves sleep quality and daytime sleepiness in Parkinson's disease: A randomized trial by Katarzyna Śmiłowska, Nienke M de Vries, Bastiaan R Bloem and Daniel J van Wamelen in Journal of Parkinson's Disease

sj-docx-2-pkn-10.1177_1877718X261468544 - Supplemental material for Blue light therapy delivered through light glasses improves sleep quality and daytime sleepiness in Parkinson's disease: A randomized trial Supplemental material, sj-docx-2-pkn-10.1177_1877718X261468544 for Blue light therapy delivered through light glasses improves sleep quality and daytime sleepiness in Parkinson's disease: A randomized trial by Katarzyna Śmiłowska, Nienke M de Vries, Bastiaan R Bloem and Daniel J van Wamelen in Journal of Parkinson's Disease
