# Supplementary material for: Blue light therapy delivered through light glasses improves sleep quality and daytime sleepiness in Parkinson's disease: A randomized trial
Source: J Parkinsons Dis. 2026 Aug 6;16(6):1201–10. doi: 10.1177/1877718X261468544 (PMC13447595; doi:10.1177/1877718X261468544)
Supplement: Supplementary file 1 [file sj-docx-1-pkn-10.1177_1877718X261468544.docx]

**Supplementary Table 2**

| **GCI scores (clinician)** | | **Blue light therapy (active treatment)** | **Red light therapy**  **(placebo)** | **p** |
| --- | --- | --- | --- | --- |
| **Week 2** | *Very much improved*  *Much improved*  *Somewhat improved*  *No change* | 46.7%  40.0%  13.3%  0.0% | 26.7%  33.3%  33.3%  6.7% | 0.363 |
| **Week 5** | *Very much improved*  *Much improved*  *Somewhat improved*  *No change* | 40.0%  53.3%  0.0%  6.7% | 0.0%  20.0%  53.3%  26.7% | **<0.001** |
