# Supplementary material for: Blue light therapy delivered through light glasses improves sleep quality and daytime sleepiness in Parkinson's disease: A randomized trial
Source: J Parkinsons Dis. 2026 Aug 6;16(6):1201–10. doi: 10.1177/1877718X261468544 (PMC13447595; doi:10.1177/1877718X261468544)
Supplement: Supplementary file 2 [file sj-docx-2-pkn-10.1177_1877718X261468544.docx]

**Supplementary Table 1**

Abbreviations: hrs: hours; min: minutes; BL: baseline; W2: week 2; W5: week 5. Level of significance adjusted for multiple testing using Bonferroni correction.
